# Extensive Thrombosis of the Inferior Vena Cava and Left Renal Vein in a Neonate

**DOI:** 10.1155/2015/569797

**Published:** 2015-06-01

**Authors:** Moez Kdous, Oussema Khlifi, Marwene Brahem, Mohamed Khrouf, Sarah Amari, Monia Ferchiou, Fethi Zhioua

**Affiliations:** Department of Obstetrics and Gynecologic Surgery, Aziza Othmana Hospital, Kasbah, 1008 Tunis, Tunisia

## Abstract

Antenatal renal vein thrombosis is a rarely described diagnostic finding, with variable consequences on kidney function. We present the case of an affected fetus, born at 35-week gestation, with intrauterine oligohydramnios and two small kidneys. A renal ultrasound carried out after birth confirmed the presence of prenatal abnormalities. Renal vein thrombosis was not diagnosed at the time. The baby died 20 days later of kidney failure, metabolic acidosis, and polypnea with severe hypotrophy. Autopsy revealed atrophied kidneys and adrenal glands. The vena cava had thrombosis occupying most of its length. The right renal vein was normal, while the left renal vein was threadlike and not permeable. Histologically, there was necrosis of the left adrenal gland with asymmetrical bilateral renal impairment and signs of ischemic and hemorrhagic lesions. A review of thrombophilia was carried out and a heterozygous mutation in Factor V was found in both the mother and the child.

## 1. Introduction

Antenatal renal vein thrombosis is a rarely described diagnostic finding, with variable consequences for kidney function. Its prevalence ranges from 2.2 to 50/100 000 births [1]. Renal vein thrombosis is particularly serious and can occur insidiously during pregnancy. It is difficult to define a group of patients at risk or a standardized approach to monitoring, surveillance, and prevention, given the small number of cases. In the antenatal period, the condition is usually unknown to sonographers and it is mainly diagnosed after birth. We present the case of an affected fetus that was born by cesarian section at 35-week gestation.

## 2. Case Presentation

The mother was a nulliparous female, with a personal history of ectopic pregnancy and epilepsy treated over the past 10 years. Her family history consisted of a paternal grandfather who had died from a pulmonary embolism at the age of 65, after cataract surgery. Her 1st and 2nd trimester ultrasounds were normal. A 3rd trimester ultrasound, performed at 33 weeks, detected oligohydramnios with severe right renal hypotrophy, compared to the left kidney. The right kidney appeared homogeneously hypoechoic in its cortex with a hyperechoic pyelovascular area. It measured 31 mm (<2nd percentile). The left kidney measured 40 mm (10th percentile), had a thinner cortex, was hyperechoic, and showed anomalies of corticomedullar differentiation with 22 highly visible pyramids. The fetus was eutrophic. An ultrasound carried out at 35 weeks revealed two small kidneys, without cysts or expansions.

After birth by cesarian section, a renal ultrasound confirmed the prenatal abnormalities with a right kidney measuring 30 mm and a left kidney measuring 27 mm. Renal vein thrombosis was not diagnosed at the time. The baby died 20 days later of kidney failure, metabolic acidosis, and polypnea with severe hypotrophy.

**Figure 1:**
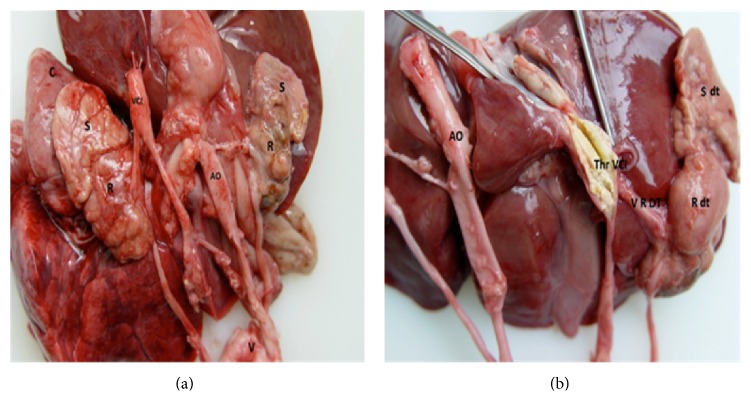
Vena cava with thrombosis occupying most of its length. Severe right renal hypotrophy, compared to the left kidney (R: kidney; S: adrenal gland; AO: aorta; VCI: inferior vena cava; R dt: right kidney; S dt: right adrenal gland).

**Figure 2:**
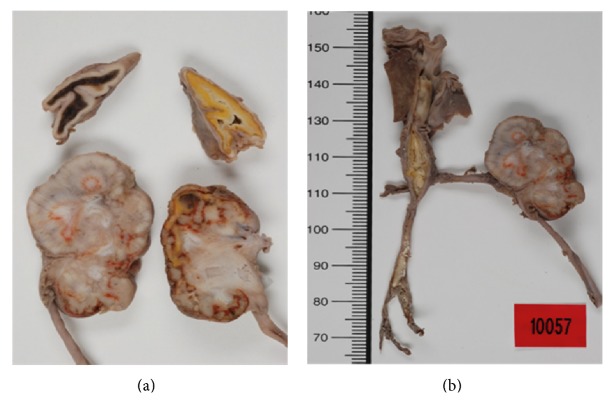
Normal right renal vein and threadlike, nonpermeable left renal vein with a white chalky content.

**Figure 3:**
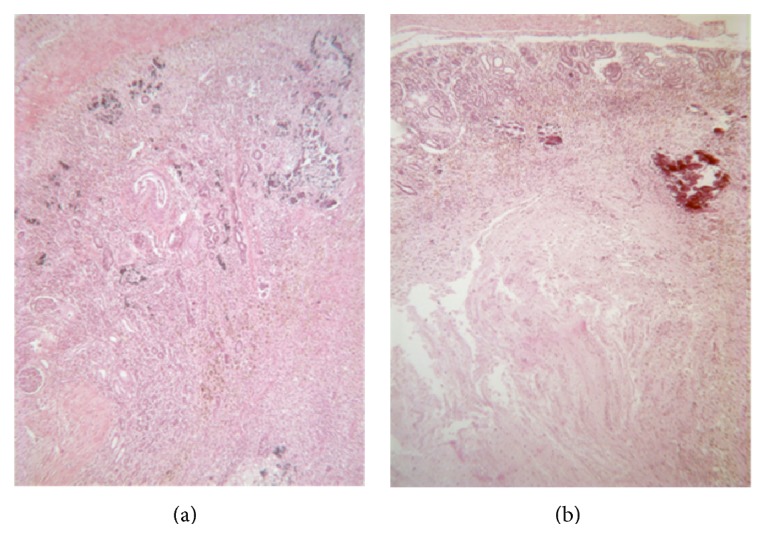
Necrosis of the left adrenal gland with asymmetrical bilateral renal impairment and signs of ischemic and hemorrhagic lesions. The renal capsule was thickened on both sides with fibrosis extending to the perirenal brown fat.

**Figure 4:**
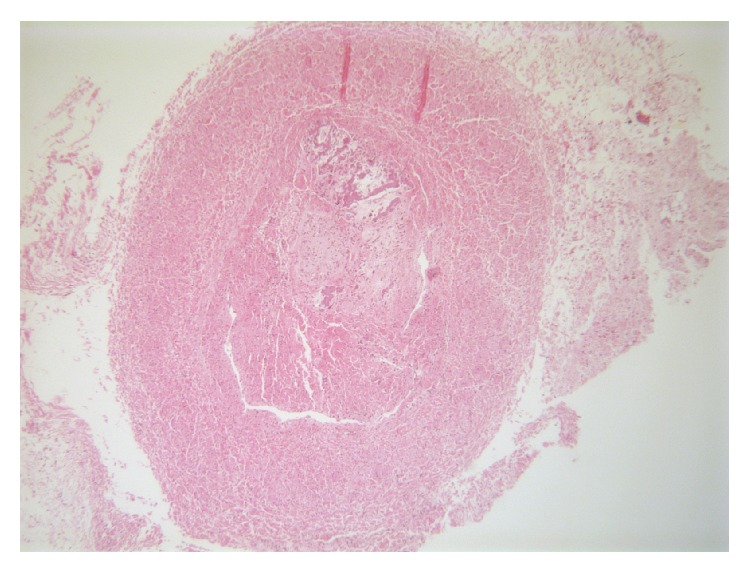
Cross section of the left renal vein showing occlusion by precalcified fibrosis, confirming that the thrombosis took place gradually.

Autopsy revealed atrophied kidneys and adrenal glands with perirenal adhesions. The vena cava had thrombosis occupying most of its length (Figure 1). The right renal vein was normal, while the left renal vein was threadlike and not permeable, with a white chalky content (Figure 2). There was no evidence of renal tract malformation or congenital renal vein defects. Histologically, there was necrosis of the left adrenal gland with asymmetrical bilateral renal impairment and signs of ischemic and hemorrhagic lesions. The renal capsule was thickened on both sides and fibrosis extended to the perirenal brown fat (Figure 3). Glomerular count was decreased for term. A cross section of the left renal vein showed occlusion by precalcified fibrosis, confirming that the thrombosis took place gradually (Figure 4).

A review of thrombophilia was carried out in the parents and the neonate including the search for Factor V or Factor II mutations, protein S deficiency, antithrombin III, and protein C, as well as a mutation in the MTHFR gene. The results revealed heterozygous Factor V Leiden in both the mother and the neonate.

## 3. Discussion

Neonatal renal vein thrombosis was first described by Rayer in 1837 and is a rare event that went undiagnosed for many decades until its discovery during surgery or postmortem. Its incidence varies from 0.5‰ in admissions to neonatal intensive care units to 0.5% in autopsy series [2]. Some cases may occur in the antenatal period. Most authors agree that thrombosis begins in the small veins of the renal parenchyma and expands towards the large venous trunks up to the renal vein or inferior vena cava. Furthermore, compression of the left renal vein by the aorta is also linked to a higher prevalence of thrombosis of the left renal vein, in its unilateral form [3]. Any maternal and/or fetal condition promoting hyperosmolarity may cause the development of renal vein thrombosis. The risk factors for thrombosis can be classified into three types: biological, amnestic, and clinical. Biological risk factors include protein C, protein S, and antithrombin III deficiencies; Factor II or Factor V mutations; hyperhomocysteinemia linked to a homozygous mutation in the MTHFR gene; homozygous sickle cell disease; anticardiolipin antibodies; and circulating lupus anticoagulant in the mother's blood which is transmitted to the fetus in utero [4–6]. Identified amnestic and clinical risk factors include caesarean section; male gender; prenatal anoxia; maternal history of thrombosis; pregnancy-induced hypertension; gestational diabetes; premature birth; dehydration; shock; and any cause of increased osmolarity. Nearly 50% of cases will demonstrate thrombophilia [7, 8]. In the case reported here, the mother and child were both heterozygous for the Factor V Leiden mutation. A heterozygous mutation is associated with a 3- to 4-fold increase in thrombotic risk, while, in the case of a homozygous mutation, the risk is multiplied 50 to 80 times [9]. As 5% of the population have the heterozygous Leiden mutation, there is no evidence that this is related to the renal vein thrombosis in our case [9].

Typical postnatal symptoms of renal vein thrombosis include an abdominal mass, bloody urine, and thrombocytopenia. The diagnosis is achieved through ultrasound. Doppler ultrasound is the gold standard to confirm renal vein obstruction and to detect its extension to the contralateral kidney, inferior vena cava, and adrenal glands. The ultrasound findings depend on the stage of thrombosis. Initially, the interlobar and interlobular furrows appear hyperechoic. Quickly, the kidney becomes globular and hyperechoic with hypoechoic pyramids, with the eventual loss of corticomedullar differentiation. Doppler (done in postnatal studies) reveals the disappearance of venous flow, an elevated resistance index in the artery, with, occasionally, the appearance of reverse flow [10, 11].

The symptoms can be difficult to identify in utero, especially as suggestive signs such as bloody urine are missing. Moreover, there can be technical obstacles (unfavorable position of the fetus, multiple pregnancies, and lack of echogenicity of some patients). There is also the possibility of false positives or spontaneous recovery. A prenatal ultrasound diagnosis can be suggested in cases of a large hyperechoic kidney, hyperechogenicity following the path of the interlobular veins, thrombus in the inferior vena cava, and Doppler indexes in the renal artery with reverse flow. There is a prognostic relation between kidney size and postnatal consequences: the larger the kidney, the worse the prognosis [12]. Patients with a family or personal history of thrombosis, thrombophilia or autoimmune disease, diabetes, fetal growth restriction, or hypotrophy should be subjected to additional ultrasounds. In these patients in particular, an extra focus on kidney examination is recommended.

Medical management of renal vein thrombosis includes aggressive hydration and anticoagulation. Nevertheless, previous studies report conflicting data regarding the benefit of anticoagulation with regard to long-term renal function, particularly in cases of bilateral renal vein thrombosis [3, 13, 14]. Thrombolytic therapy may be considered in cases of bilateral renal vein thrombosis, especially if there is concomitant renal failure [15]. Definitive surgical treatment consists of nephrectomy and thrombectomy on a nonurgent basis, provided there is no caval extension and obstruction. Thrombectomy for bilateral renal vein thrombosis with caval involvement and obstruction has been described once before, but with subsequent unilateral nephrectomy [16]. Recently, Lee et al. [4] showed that bilateral renal vein thrombosis can be successfully managed with early surgical thrombectomy without the need for nephrectomy, thereby avoiding the significant morbidity associated with infant dialysis and renal transplantation. Successful restoration of renal function after surgical thrombectomy in his patient illustrates an encouraging treatment option [4]. However, the relatively small number of reported cases and lack of prospective trials have opened up debate regarding the best way to manage this condition.

To date, there are still many unresolved issues regarding antenatal renal vein thrombosis. There are still large discrepancies in diagnostic possibilities and prognosis. It would be ideal to keep a register of all cases of antenatal renal vein thrombosis, from different obstetric teams. A standardized approach for monitoring, surveillance, and prevention in subsequent pregnancies is yet to be defined. It is essential to learn how to diagnose this condition, as is it necessary to update obstetric ultrasound books and teaching methods for obstetricians.

## References

[1] Bökenkamp A., von Kries R., Nowak-Göttl U., Göbel U., Hoyer P. F. (2000). Neonatal renal venous thrombosis in Germany between 1992 and 1994: epidemiology, treatment and outcome. *European Journal of Pediatrics*.

[2] Schmidt B., Andrew M. (1995). Neonatal thrombosis: report of a prospective Canadian and international registry. *Pediatrics*.

[3] Lau K. K., Stoffman J. M., Williams S. (2007). Neonatal renal vein thrombosis: review of the english-language literature between 1992 and 2006. *Pediatrics*.

[4] Lee S., Ananth P., Boyd T., Esrick E., Kim H. B. (2014). Successful surgical thrombectomy for neonatal IVC and bilateral renal vein thrombosis. *Journal of Pediatric Surgery Case Reports*.

[5] Kosch A., Kuwertz-Bröking E., Heller C., Kurnik K., Schobess R., Nowak-Göttl U. (2004). Renal venous thrombosis in neonates: prothrombotic risk factors and long-term follow-up. *Blood*.

[6] Smorgick N., Herman A., Wiener Y., Halperin R., Sherman D. (2007). Prenatal thrombosis of the inferior vena cava and the renal veins. *Prenatal Diagnosis*.

[7] Kuhle S., Massicotte P., Chan A., Mitchell L. (2004). A case series of 72 neonates with renal vein thrombosis. Data from the 1-800-NO-CLOTS registry. *Thrombosis and Haemostasis*.

[8] Zigman A., Yazbeck S., Emil S., Nguyen L. (2000). Renal vein thrombosis: a 10-year review. *Journal of Pediatric Surgery*.

[9] Vern T. Z., Alles A. J., Kowal-Vern A., Longtine J., Roberts D. J. (2000). Frequency of factor V(Leiden) and prothrombin G20210A in placentas and their relationship with placental lesions. *Human Pathology*.

[10] Hibbert J., Howlett D. C., Greenwood K. L., Macdonald L. M., Saunders A. J. S. (1997). The ultrasound appearances of neonatal renal vein thrombosis. *British Journal of Radiology*.

[11] Kraft J. K., Brandão L. R., Navarro O. M. (2011). Sonography of renal venous thrombosis in neonates and infants: can we predict outcome?. *Pediatric Radiology*.

[12] Winyard P. J. D., Bharucha T., De Bruyn R. (2006). Perinatal renal venous thrombosis: presenting renal length predicts outcome. *Archives of Disease in Childhood: Fetal and Neonatal Edition*.

[13] Messinger Y., Sheaffer J. W., Mrozek J., Smith C. M., Sinaiko A. R. (2006). Renal outcome of neonatal renal venous thrombosis: review of 28 patients and effectiveness of fibrinolytics and heparin in 10 patients. *Pediatrics*.

[14] Brandão L. R., Simpson E. A., Lau K. K. (2011). Neonatal renal vein thrombosis. *Seminars in Fetal and Neonatal Medicine*.

[15] Jaako Dardashti V., Békássy Z. D., Ljung R. (2009). Successful thrombolysis of neonatal bilateral renal vein thrombosis originating in the IVC. *Pediatric Nephrology*.

[16] Clark A. G. B., Saunders A., Bewick M., Haycock G., Chantler C. (1985). Neonatal inferior vena cava and renal venous thrombosis treated by thrombectomy and nephrectomy. *Archives of Disease in Childhood*.

