# Corrigendum to “Extensive Thrombosis of the Inferior Vena Cava and Left Renal Vein in a Neonate”

**DOI:** 10.1155/2016/3612685

**Published:** 2016-09-19

**Authors:** Moez Kdous, Oussama Khlifi, Marwene Brahem, Mohamed Khrouf, Sarah Amari, Monia Ferchiou, Fethi Zhioua

**Affiliations:** Department of Obstetrics and Gynecologic Surgery, Aziza Othmana Hospital, Kasbah, 1008 Tunis, Tunisia

After the publication of our article titled “Extensive Thrombosis of the Inferior Vena Cava and Left Renal Vein in a Neonate” [1], a reader noted that in the Discussion (treatment paragraph) we have not taken into consideration some references when we discussed surgical treatment of renal vein thrombosis, especially when we talked about thrombectomy.

After discussion with coauthors, we have decided to add one reference to our paper, reference number 17: A. Moaddab, A. A. Shamshirsaz, R. Ruano et al., “Prenatal diagnosis of renal vein thrombosis: a case report and literature review,”* Fetal Diagnosis and Therapy*, 2015. This will be added in Discussion, after reference number 16.

The edited part of the paper is as follows.

## 3. Discussion

Medical management of renal vein thrombosis includes aggressive hydration and anticoagulation. Nevertheless, previous studies report conflicting data regarding the benefit of anticoagulation with regard to long-termrenal function, particularly in cases of bilateral renal vein thrombosis [3, 13, 14].

Thrombolytic therapy may be considered in cases of bilateral renal vein thrombosis, especially if there is concomitant renal failure [15]. Definitive surgical treatment consists of nephrectomy and thrombectomy on a nonurgent basis, provided there is no caval extension and obstruction. Thrombectomy for bilateral renal vein thrombosis with caval involvement and obstruction has been described once before, but with subsequent unilateral nephrectomy [16, 17].

Recently, Lee et al. [4] showed that bilateral renal vein thrombosis can be successfully managed with early surgical thrombectomy without the need for nephrectomy, thereby avoiding the significant morbidity associated with infant dialysis and renal transplantation.
